# A good response of refractory mantel cell lymphoma to haploidentical CAR T cell therapy after failure of autologous CAR T cell therapy

**DOI:** 10.1186/s40425-019-0529-9

**Published:** 2019-02-21

**Authors:** Tongjuan Li, Yuanyuan Zhang, Dan Peng, Xia Mao, Xiaoxi Zhou, Jianfeng Zhou

**Affiliations:** 1 Department of Hematology, Tongji Hospital, Tongji Medical College, Huazhong University of Science and Technology, Wuhan, Hubei China; 2 Department of Nuclear medicine, Tongji Hospital, Tongji Medical College, Huazhong University of Science and Technology, Wuhan, Hubei China

**Keywords:** Haplo-identical CAR T cell therapy, Mantel cell lymphoma

## Abstract

**Background:**

The aggressive form of Mantle cell non-hodgkin B cell lymphoma (MCL) has a dismal prognosis. Dual targeting BTK and BCL2 with ibrutinib and venetoclax has improved outcomes in MCL patients who were predicted not to respond to conventional therapy, but it is unlikely to be curative. Chimeric antigen receptor-modified T (CAR T) cells exhibit very effective function in elimination of relapsed/refractory B-cell lymphoid malignancies, we investigated their use in a patient with relapsed MCL.

**Case presentation:**

Here, we report a case of a refractory MCL in a patient who had relapsed after conventional chemotherapy and autologous CAR T cell therapy. The patient received multiple molecularly targeted therapies, including targeting BTK and BCL2, and haplo-identical CAR T (haplo-CAR T) cells from her daughter without previous allo-hematopoietic stem cell transplantation. Haplo-CAR T cells could effectively proliferate in vivo and had a clinically significant antitumor activity without serious side effects. The patient achieved a partial remission, with minimal residual disease.

**Conclusions:**

This case suggests that haplo-CAR T cell therapy can be effective in controlling lymphoma that failed to respond to autologous CAR T cell therapy and overcome limitation of autologous CAR T cells, thus may be one possible regimen before the era of off-the-shelf “universal” CAR T cell therapy.

**Trial registration:**

ChiCTR-OPN-16008526. http://www.chictr.org.cn/showproj.aspx?proj=13798; ChiCTR1800019385. http://www.chictr.org.cn/showproj.aspx?proj=32805; ChiCTR1800019449. http://www.chictr.org.cn/showproj.aspx?proj=32778.

**Electronic supplementary material:**

The online version of this article (10.1186/s40425-019-0529-9) contains supplementary material, which is available to authorized users.

## Introduction

Mantle cell lymphoma (MCL) is a type of non-Hodgkin B cell lymphoma with a distinctive molecular marker cyclin D1 that is constitutively overexpressed in almost all cases. MCL can be both indolent or aggressive, in either case it responds poorly to chemotherapy and consequently the aggressive form has a dismal prognosis assessed by incorporating Ki-67 proliferation index and Mantle Cell International Prognostic Index scores. An orally administered, irreversible inhibitor of Bruton’s tyrosine kinase (BTK), ibrutinib, is effective at arresting the progression of MCL [1] as is a highly selective BCL2 inhibitor, venetoclax (ABT-199, Venclexta™) [2]. Dual targeting BTK and BCL2 with ibrutinib and venetoclax has increased complete response rate compared with ibrutinib monotherapy in MCL patients but it is unlikely that this combination therapy will lead to a long term cure of the disease [3]. Chimeric antigen receptor-modified T (CAR T) cells are highly effective in the treatment of common pre-B cell acute lymphoblastic leukemia and are currently under assessment for the treatment of relapsed/refractory B-cell lymphoid malignancies, such as diffuse large-B-cell lymphoma (DLBCL) [4], follicular lymphoma [5]. In MCL, their use has had missed results [6]. Here, we report a case of a refractory MCL receiving multiple molecularly targeted therapies and haplo-identical CAR T cells from her daughter and achieving a partial remission with only minimal residual disease.

## Case presentation

### The medical history

A 40-year-old female patient had been diagnosed as classical Mantle cell lymphoma (MCL) at stage IV B with deletion of TP53 gene by lymph node biopsy in local hospital at September, 2017. The immumohistochemical staining results were as follows: CD20(+), PAX5(+), CD79a(+/−), CD5(+), CD21(+), CD23(+), CycIin-D1(+), Ki-67(30%), CD43(mild+), BCL-2(+), BCL-6(+), SOX11(partial +), and molecules including CD2, CD3, CD7, CD10, TIA1, GrB and TdT were negative. EBV was undetectable by in situ hybridization. She had received first and second line chemotherapy including R-CHOP, R-DHAP and R-VCOP, but had progressive disease. Only the combination of ibrutinib and rituximab (IR) resulted in a transient partial remission. In March 2018, she came to our hospital for CAR T cell therapy, a clinical trial of sequential infusion of CART19 (or CART20) and CART22 expressing murine scFv of anti-CD19, anti-CD20 and anti-CD22 in combination with CD28 and 4-1BB costimulatory domains, and CD3ζ signaling domain (ClinicalTrials.gov number ChiCTR-OPN- 16008526; ChiCTR1800019385 and ChiCTR1800019449).

### Clinical findings

When she was admitted to our hospital, she had a fever, severe dyspnea, and hypoxemia with the lowest SpO2 of 80%. Systemic edema, superficial lymphadenopathy and splenomegaly (reaching her pelvic cavity) were found by physical examination. The lymph nodes were about 3 cm in diameter, like beads-on-string. The number of leukocytes was 71.97*10^9/L in the peripheral blood, and the level of serum lactate dehydrogenase (LDH) was elevated (up to 1619 U/L). Follow-up FDG-PET/CT (Positron Emission Tomography-Computed Tomography) showed swollen lymph nodes throughout the body with increased metabolism, the maximum standard uptake value was 5.7. The maximum standard uptake value of splenomegaly was 6.2. The bone metabolism was hyperactive, and the maximum standard uptake value was 7.5. Furthermore, the lungs and bilateral adrenal were also infiltrated by lymphoma (max Deauville score 5) (Fig. 3a). 66 and 63% of lymphoblasts could be found in the peripheral blood and bone marrow smear, respectively. Her complex karyotype was 43, X, −X, − 9,-10,t(11;14)(q13;q32), del(13)(q12q22). A deletion of exon 7 of TP53 gene that induces frameshift (c.756delC,p.R253Pfs*92), and a nonsense mutation of the exon 34 of NOTCH2 gene (c.7242C > A, p.Y2414*;C.7176 T > G,p.Y2392*) were found by next-generation sequencing (NGS). The patient was high risk evaluated by MIPI.

### Autologous CAR T cell therapy

**Fig. 1:**
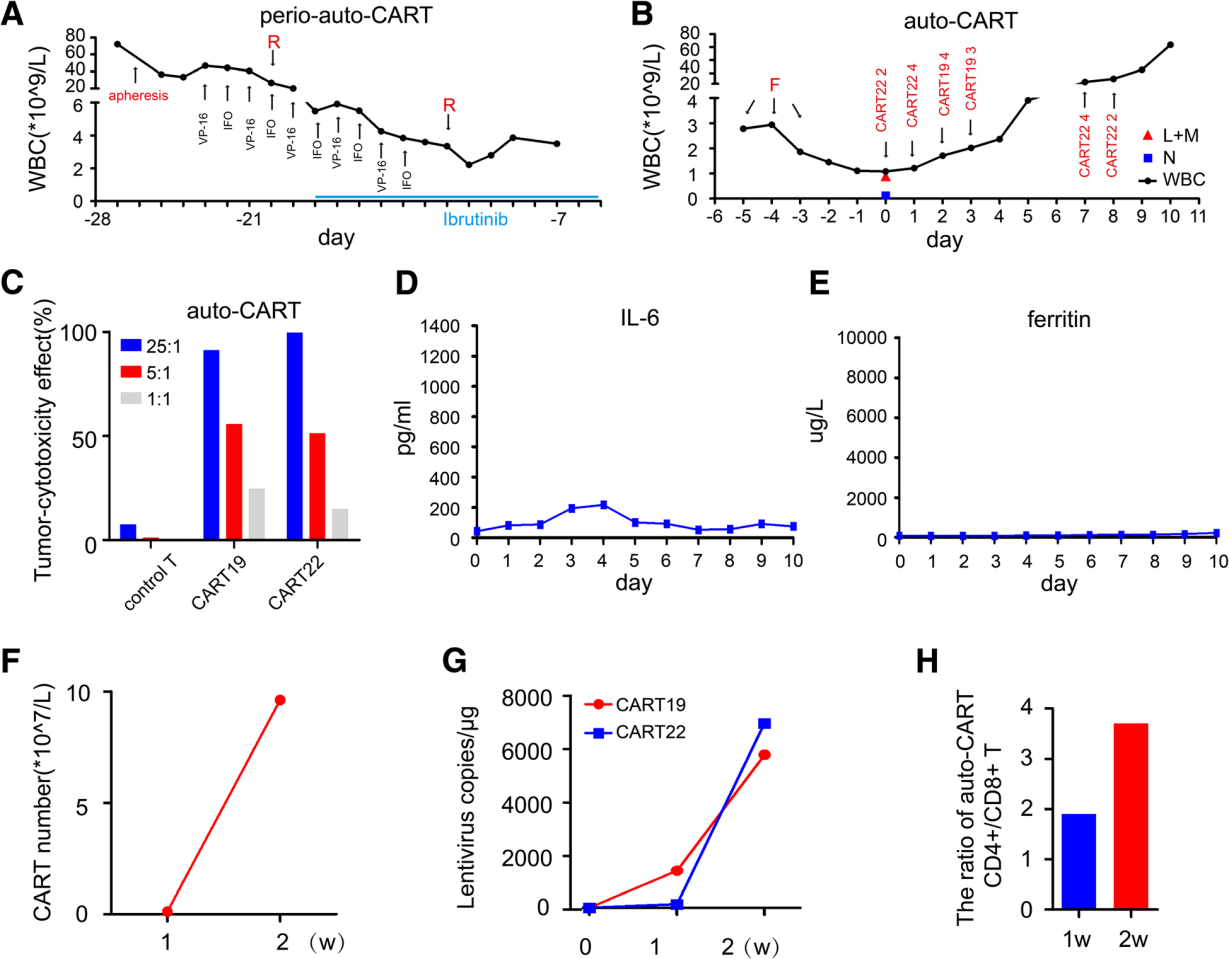
The failure of autologous CAR T cell therapy. **a** Treatments and the white blood cell changes during perio-autologous CAR T cell therapy. The first infusion day of autologous CAR T cells was as day 0, apheresis was used at day − 26 and rituximab (R) 375 mg/m^2^ was used at day − 20 and − 12 before infusion of CAR T cells (red font). Etoposide (VP-16) 100 mg and ifosfamide (IFO) 1 g were used alternately between day − 23 and day − 14 before infusion (black font). The treatment with ibrutinib 560 mg daily was between day − 18 and day − 5 before infusion (blue line). **b** Treatments and the white blood cell changes during autologous CAR T cell therapy. The red triangle represents the number of lymphocytes (L) and monocytes (M), blue square represents the level of neutrophile granulocytes (N). Fludarabine (F) 25 mg/m^2^ was used on day − 5 and − 3 before infusion (red font). Autologous CART22 was infused on day 0, day 1, day 7 and day 8, while autologous CART19 was infused on day 2 and day 3. **c** In vitro tumor-cytotoxicity effect of autologous CART19 and CART22 cells at an effector/target ratio of 25:1, 5:1 and 1:1 respectively. **d** and **e** Levels of IL-6 and ferritin during autologous CAR T cell therapy. **f** and **g** CAR T cell number and copies of lentivirus-containing CAR in the peripheral blood detected by flow cytometry and ddPCR after autologous CAR T cell therapy. **h** The ratio of CD4+/CD8+ T cells in the peripheral blood was 1.9 on day 7 and 3.7 on day 14

In view of the patient’ s poor clinical status, we removed the excess tumor cells by apheresis and isolated primary T lymphocytes from peripheral blood to urgently prepare anti-CD19 chimeric antigen receptor T cells (CART19) and anti-CD22 chimeric antigen receptor T cells (CART22). The patient was conditioned with VP-16 100 mg and ifosfamide 1 g were used alternately, combined with rituximab 375 mg/m^2^ every week together with ibrutinib 560 mg daily to halt the tumor progression (Fig. 1a). The line chart showed that rituximab had a transient tumor-cytotoxic effect. About 20 days later, the patient’s general condition improved. Edema of her face and lower limbs had appreciably diminished with shrank splenomegaly to the horizontal of the umbilicus. Then, she received lympho-depleting chemotherapy with fludarabine (25 mg/m^2^ on day-5~ − 3) without cyclophosphamide, as she had been treated with IFO before. Autologous CART22 (1.2*10^7 cells/kg) was infused on day 0 and day + 1, followed by CART19 (7*10^6 cells/kg) on day + 2 and day + 3 (Fig. 1b). The autologous CAR T cells proliferated in vitro and the tumor-cytotoxic effect of CART22 and CART19 was up to 99.34 and 99.01% at an effector/target ratio of 25:1 with 65.1 and 32.4% infection efficiency, respectively (Fig. 1c). At the first day of infusion, the WBC declined to 1.08*10^9/L with 0.13*10^9/L neutrophile granulocytes, 0.59*10^9/L lymphocytes and 0.3*10^9/L monocytes. She only had a mild fever and 1 grade cytokine-release syndrome (CRS), manifested as mild elevated interleukin-6 and unchanged ferritin (Fig. 1d and e). On day + 7 after autologous CAR T cell therapy, the disease significantly progressed, as the white blood cell count increased sharply, reaching 63.61*10^9/L on day + 9 in the peripheral blood, along with enhanced LDH up to 959 U/L. Despite this, the CART22 cell product was infused again on day + 7 and day + 8, which did not alter the disease progression (Fig. 1b). Moderate edema of her face and both lower limbs reappeared, and the enlarged spleen reached pelvic cavity again. These indicated that autologous CAR T cell therapy failed to halt the disease progression in spite of increased number of CAR T cells and copies of CART19 and CART22 in the blood at 2 weeks after first CAR T cell fusion (Fig. 1f and g). At this time (day + 14) the ratio of CD4+/CD8+ T cells, including CAR T cells, in the peripheral blood was 3.7, a significant rise over the normal expected ratio (Fig. 1h). The detail of the process of treatment before and after autogenous CAR T cell therapy and her responses was summerized in Additional file 1: Table S2.

### Haplo-identical CAR T cell therapy

**Fig. 2:**
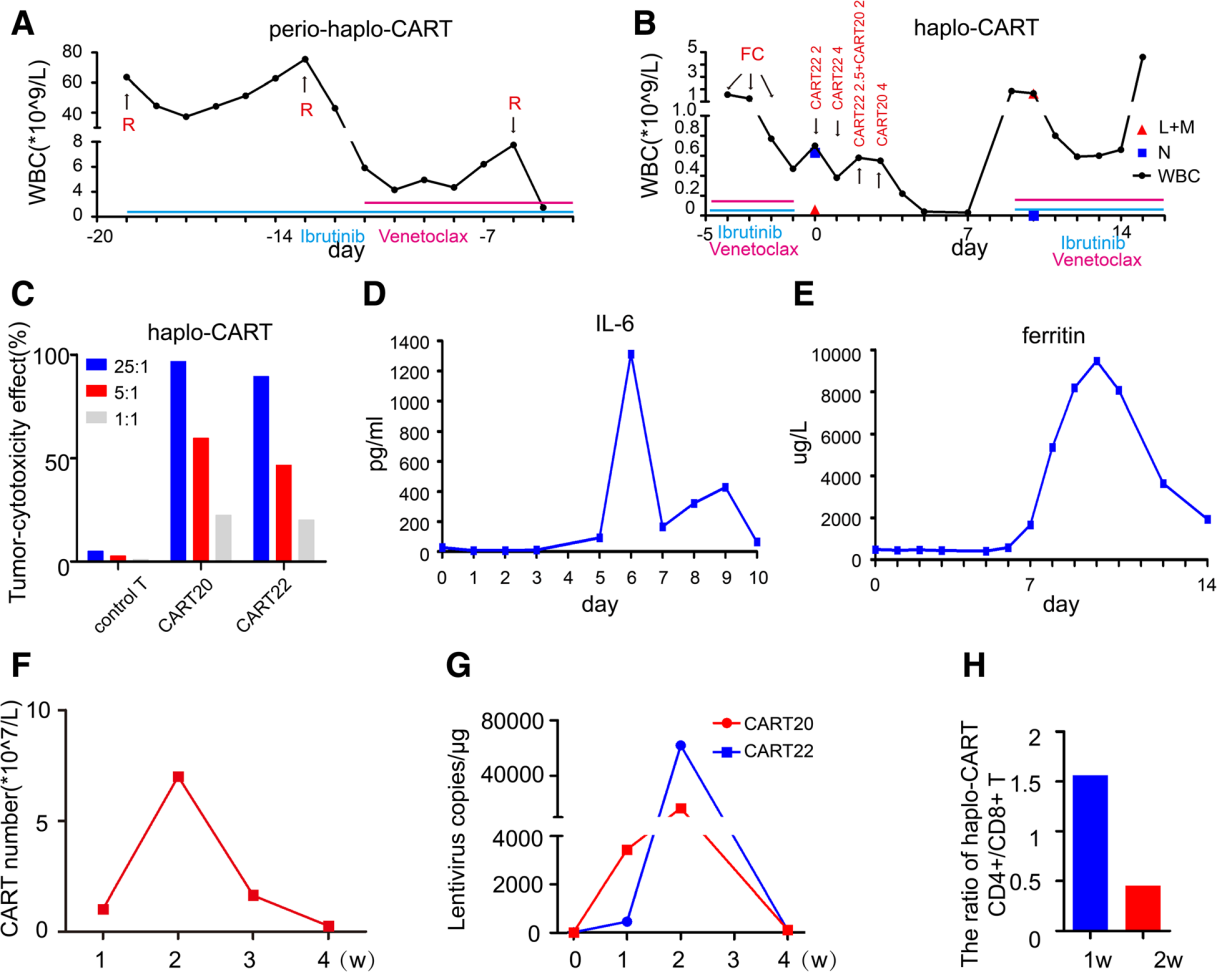
A response to haplo-CAR T cell therapy. **a** Treatments and the white blood cell changes during perio-haplo-CAR T cell therapy. The first infusion day of haplo-identical CAR T cells was as day 0, rituximab (R) 375 mg/m^2^ was used at day − 19 and 500 mg/m^2^ at day − 13 and − 6 before infusion (red font). Venetoclax 400 mg daily was used from day − 11 to day − 1 before infusion (red line). The treatment with ibrutinib 560 mg daily lasted from day − 19 to day − 1 before infusion (blue line). **b** Treatments and the white blood cell changes during haplo-CAR T cell therapy. The red triangle represents the number of lymphocytes (L) and monocytes (M), blue square represents the level of neutrophile granulocytes (N). Fludarabine (F) 25 mg/m^2^ and cyclophosphamide (C) 20 mg/kg was used on day − 4 and − 2 before infusion (red font). Haplo-identical CART22 cells were infused from day 0 to day 2, while haplo-identical CART20 cells were infused on day 2 and day 3. From day 9, ibrutinib (red line) and venetoclax (blue line) were used again. **c** In vitro tumor-cytotoxicity effect of haplo-identical CART20 and CART22 cells at an effector/target ratio of 25:1, 5:1 and 1:1 respectively. **d** and **e** Levels of IL-6 and ferritin after haplo-CAR T cell therapy. **f** and **g** CAR T cell number and copies of lentivirus-containing CAR in the peripheral blood after haplo-CAR T cell therapy. **h** The ratio of CD4+/CD8+ T cells in the peripheral blood was 1.6 at day + 7 and 0.4 at day + 14

**Fig. 3:**
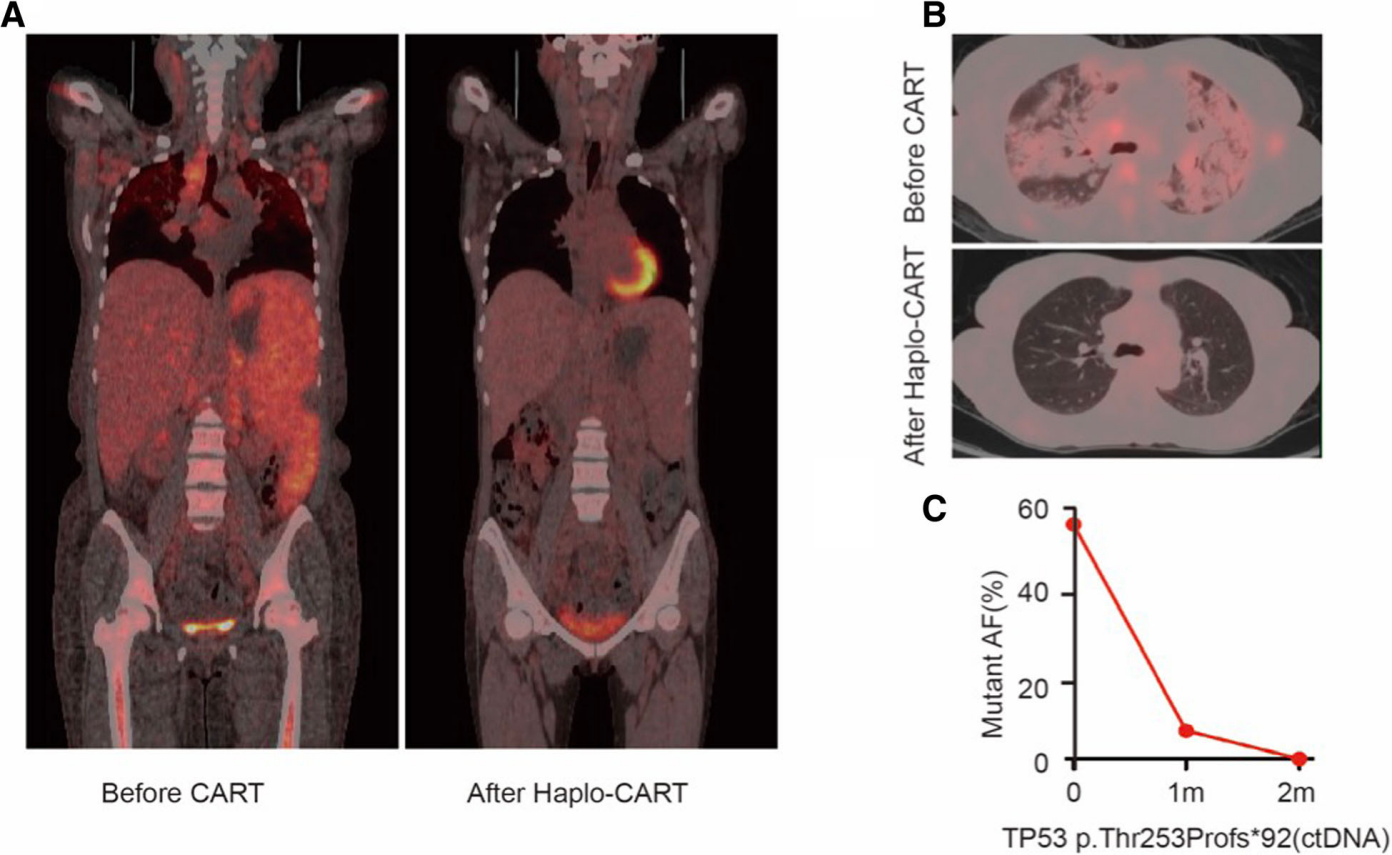
PET/CT and liquid biopsy monitoring the efficacy of haplo-CAR T cell therapy. **a** Whole body PET/CT image before CART therapy and 45 days after haplo-CAR T cell therapy. **b** Lung slice of tumor infiltration before CAR T cell therapy and 45 days after haplo-CAR T cell therapy. **c** The mutant percent of TP53 gene p.Thr253Profs*92 in circulating tumor DNA by liquid biopsy before CAR T cell therapy, in 1 and 2 months after haplo-CAR T cell therapy

In the presence of relapsing disease, we tried to repeat the therapy using haplo-identical T cells derived from the patients’ daughter transfected with the anti-CD20 and anti-CD22 CAR constructs to generate haplo-identical CART20 and CART22 cells respectively. Before haplo-CAR T cell therapy, we pre-conditioned the patient with rituximab and ibrutinib, combined with BCL-2 inhibitor venetoclax. The efficacy of rituximab was transient although the addition of venetoclax resulted in a significant drop in the white cell count (Fig. 2a). Subsequently she was conditioned with a standard lympho-depleting chemotherapy regimen consisting of fludarabine (25 mg/m^2^) and cyclophosphamide (20 mg/kg) on day − 4 ~ − 2 (Fig. 2b). This was associated with a further reduction in the size of her spleen and a drop in her peripheral WBC to 0.7*10^9/L with 0.63*10^9/L neutrophile granulocytes, 0.04*10^9/L lymphocytes and 0.02*10^9/L monocytes (Fig. 2b). Again, the haplo-CART cells could proliferate in vitro and the tumor-cytotoxic effect of CART22 and CART20 was up to 89.41 and 96.55% at an effector/target ratio of 25:1 with 49.7 and 71.2% infection efficiencies, respectively (Fig. 2c). The infusion of haplo-CAR T cells was performed as follows: 8.5*10^6 cells/kg of CART22, divided into three infusions on day 0 ~ + 2, followed by 6*10^6 cells/kg CART20, divided into two infusions on day + 2 and day + 3. On day + 10 after haplo-CAR T cell infusion, the WBC increased to 1.67*10^9/L with 0.06*10^9/L neutrophile granulocytes, and 1.42*10^9/L lymphocytes and 0.15*10^9/L monocytes (Fig. 2b). She had a persistent high temperature (maximum 39.4 °C) from day + 4 to day + 10, accompanied with obvious chest distress and facial swelling. Her plasma IL-6 concentration was 48.7 times higher than its base level, its peak reached 1314 pg/mL at day 6, and ferritin was 19.4 times higher and its peak reached 9474 μg/L at day + 10 (Fig. 2d and e), suggesting that this was a cytokine release syndrome rather than a reaction to her haplo-identical T cells. At day + 6 methylprednisolone was used to control her inflammatory reaction. Her NT-proBNP reached 8020 pg/mL and continuous renal replacement therapy was required to support her during this period. After 7 days of high fever, the patient’s general condition improved. Physical examination revealed that superficial lymph nodes had shrunk, and the spleen was palpable only 3 cm below the left costal margin. The ultrasonography indicated several splenic infarctions about 2 cm in diameter and the spleen pachydiameter was 5.4 cm. CAR T cell number and the copies of CART22 and CART20 increased dramatically and reached the peak at the second week (Fig. 2f and g). This detection time was 11 days from the last infusion of haplo-CAR T cells on day + 3. In addition, the ratio of CD4+/CD8+ T cell in the peripheral blood was significantly lower (0.4) than normal (proportional inversion) on +day 14 after haplo-CAR T cell infusion (Fig. 2h). At this time point, we were unable to detect the presence of minimal residual disease by flow cytometry and quantitative-PCR in the bone marrow. One month later, evaluation of haplo-CAR T-therapy revealed that the disease was in sustained remission. FDG-PET/CT on day + 45 was unable to detect any significant disease (max Deauville score 4), and the infiltration of tumor cells into lungs vanished (Fig. 3a and b). We next assessed the presence of minimal residual disease using next generation sequencing to monitor circulating tumor DNA. We were able to detect the presence of p.Thr253Profs*92 mutation in the TP53 gene but this had significantly reduced to a near undetectable level (Fig. 3c). Ten days after haplo-CART cell infusion, she began to be treated with ibrutinib and venetoclax again as a maintenance therapy, as a bridge to a formal haplo-identical HSCT from her daughter. The detail of the process of treatment before and after haplo-indentical CAR T cell therapy and her responses was summerized in Additional file 1: Table S3.

## Discussion

Here, we present a patient with primary progressive MCL that failed to respond to a combination of autologous CAR T cell therapies. In desperation we repeated the therapy using halpo-identical CART cell therapy that was able to induce a near complete remission with minimal residual disease. This is the first report demonstrating the anti-lymphoma efficacy of haplo-CAR T cell therapy in the absence of previous allo-HSCT. Although the patient became profoundly sick with multi-organ failure associated with a significantly elevated IL-6 consistent with a cytokine release syndrome, we did not see any clinical evidence of graft versus host disease or rejection of the host bone marrow by the donor CART cells. Furthermore, her symptoms were successfully controlled using cortico-steroids.

The different response to auto- and haplo-CAR T cell therapies may be due to several factors. First, we used slightly different targets as the original auto-CAR T cells were directed against CD19 and CD22 and the haplo-CAR T cells were directed against CD20 and CD22. The change in strategy was in part chosen by the relatively good response to rituximab. However, it is of note that all three targeted antigens were all expressed both before and after CAR T cell therapies (Additional file 1: Figure S1), and this is unlikely to be a significant reason for the difference.

The second important difference between auto- and haplo-CAR T cell therapies was the patient’s tumor burden before infusions. The total number of lymphocytes and monocytes in peripheral blood, used to assess tumor burden, were 8.9*10^8/L and 6*10^7/L before auto- and haplo-CAR T cell infusion, respectively. Ten times higher tumor burden might have stronger immune suppressive effects by releasing inhibitory cytokines TGF-β and IL-10 to hamper cytotoxic function of auto-CAR T cells and inducing differentiation of regulatory T cells (Tregs) [7]. Our results showed that the ratio of CD4+/CD8+ T cells (including CAR T cells) in peripheral blood was higher after auto-CAR T cell therapy, but the ratio was lower after haplo-CAR T cell therapy(Fig.1h and 2h). It is possible that more infused auto-CAR T cells in such an inhibitory microenvironment of higher tumor burden differentiated into CD4+ Tregs, but expansion of CD8+ cytotoxic T cells was suppressed, unlike infused haplo-CAR T cells where the CD8+ T cell was a main proliferated subpopulation. Therefore, before CAR T cell therapy, we should minimize the tumor burden and raise the ratio of CAR T cells to tumor burden, increasing effector/target ratio and efficacy of CAR T cell therapy.

The third difference is the source of T cells. An obvious advantage of haplo-CAR T cells is T cells from healthy donor, which can ensure their quality and quantity, in contrast to autologous CAR T cells which are often insufficient in number by impaired ex vivo expansion and poor quality [8, 9]. Consistently, our results showed that the copy number of haplo-CAR T cells was significantly higher than the auto-CAR T cells. Other groups have reported that the clinical response of CLL to CAR T cell therapy is influenced by T cell–intrinsic attributes [10]. CAR T cells from complete-responding patients with CLL were enriched in memory-related genes, including IL-6/STAT3 signatures, whereas T cells from non-responders upregulated programs involved in effector differentiation, glycolysis, exhaustion and apoptosis. T cells from leukemia-bearing mice presented increased expression of inhibitory co-receptors including programmed cell death protein 1 (PD1), Tim3 and LAG3, and introduction of a CAR into these T cells failed to fully reverse poor in vivo function [11]. However, T cells from a haplo-source of healthy donor were not affected by leukemia before infusion and may express lower levels of the inhibitory molecules than T cells from the lymphoma patient. In addition, T cells from healthy donor have normal functions and may contain less regulatory T cells than T cells from the tumor patient.

One of factors influencing the efficacy of CAR T cell therapy is multiple molecular targeted therapies including rituximab, ibrutinib and venetoclax. As the patient had significant but transient response to rituximab, we used rituximab to control tumor burden during preparation of CAR T cells. Although residual serum rituximab may compete with CART20 for binding to CD20, it has been reported that the activity of CD20-targeted CAR T cells is preserved in vivo at clinically relevant levels of rituximab [12]. Ibrutinib, a small molecule drug that binds permanently to a protein-Bruton's tyrosine kinase (BTK), was used until the infusion of haplo-CAR T cells. A previous research in vitro and in a mouse xenograft MCL model found that addition of ibrutinib to CAR T cells improves responses against MCL [13]. Fraietta, J.A., et al. [14] found that ibrutinib improved the expansion and function of CAR T cells ex vivo and in vivo, and decreased expression of the immunosuppressive molecule PD1 on T cells and on tumor cells. Venetoclax, targeting BCL2, was used after the failure of auto-CAR T cell therapy. The previous studies have revealed that small-molecule inhibitors of the BCL2 family potentiate the efficacy of CAR T cells by sensitizing tumor cells to apoptosis [8]. Importantly, venetoclax induces apoptosis in a TP53-independent manner [15] and is suggested as an optimal partner with ibrutinib by pharmacological and protein profiling [16]. This was also the possible reason for the success of haplo-CART therapy. These targeted therapies not only controlled disease progression, but also did not affect the function of CAR T cells, suggesting a potential synergistic benefit. Combining CAR T cell therapy and such supplementary tactics represents a promising two-pronged therapeutic strategy in patients with MCL or other types of B-cell lymphoma.

Finally, tumor cells have the ability to escape the immune surveillance through genetic or epigenetic changes and become tolerant to the cytotoxic activity of their own T cells [17]. But the donor T cells can eliminate tumor cells by recognizing tumor-specific or recipient-specific antigens [18]. According to our data, of haplo-CAR T cells administered to the patient, 41.4% of T lymphocytes were her daughter’s non-CAR-transduced T cells that may exert graft-versus- lymphoma activity. This is also the possible reason for the success of haplo-CAR T cell therapy. This case suggests that haplo-identical CAR T cell therapy can be effective in controlling lymphoma that failed to respond to autologous CAR T cell therapy and help overcome limitation of autologous CAR T cells, thus may be one possible regimen before the era of off-the-shelf “universal” CAR T cell therapy.

## Additional file

Additional file 1: **Method S1.** Preparation of CAR-T cells. **Method S2.** Cell surface staining and flow cytometry. **Method S3.** Copy number of CAR and cellular kinetics parameters. **Method S4.** Detection of plasma cytokines. **Figure S1.** The expression of CD19, CD20 and CD22 on lymphoma cells. A and B showed the expression of CD19, CD20 and CD22 in lymphoma cells before auto-CAR T cell therapy. C and D showed the expression of CD19, CD20 and CD22 in lymphoma cells before haplo-CAR T cell therapy. **Table S1.** Sequences of primers used in ddPCR. **Table S2.** Timeline of treatment and efficacy of auto-CAR T. **Table S3.** Timeline of treatment and efficacy of haplo-CAR T cell therapy. (DOCX 151 kbf)
