# Supplementary material for: A good response of refractory mantel cell lymphoma to haploidentical CAR T cell therapy after failure of autologous CAR T cell therapy
Source: J Immunother Cancer. 2019 Feb 21;7:51. doi: 10.1186/s40425-019-0529-9 (PMC6383265; doi:10.1186/s40425-019-0529-9)
Supplement: Supplementary file 1 — Method S1. Preparation of CAR-T cells. Method S2. Cell surface staining and flow cytometry. Method S3. Copy number of CAR and cellular kinetics parameters. Method S4. Detection of plasma cytokines. Figure S1. The expression of CD19, CD20 and CD22 on lymphoma cells. A and B showed the expression of CD19, CD20 and CD22 in lymphoma cells before auto-CAR T cell therapy. C and D showed the expression of CD19, CD20 and CD22 in lymphoma cells before haplo-CAR T cell therapy. Table S1. Sequences of primers used in ddPCR. Table S2. Timeline of treatment and efficacy of auto-CAR T. Table S3. Timeline of treatment and efficacy of haplo-CAR T cell therapy. (DOCX 151 kbf) [file 40425_2019_529_MOESM1_ESM.docx]

**A Good Response of Refractory Mantel Cell Lymphoma to Haploidentical CAR T Cell Therapy after Failure of Autologous CAR T Cell Therapy**

Tongjuan Li^1^*，Yuanyuan Zhang^1^*,Dan Peng^2^ , Xia Mao^1^, Xiaoxi Zhou^1&^，Jianfeng Zhou^1&^

^1^Department of Hematology and ^2^Department of Nuclear medicine, Tongji Hospital, Tongji Medical College, Huazhong University of Science and Technology, Wuhan, Hubei, China

**Supplementary Data**

**Methods**

**Supplementary Figure 1**

**Supplementary table 1**

**Supplementary table 2**

**Methods:**

**Preparation of CAR-T cells**

Lymphocytes were obtained from the patient (or her daughter) through lymphocyte apheresis under protocols approved by the Ethic Committee of Tongji Hospital, Tongji Medical College, Huazhong University of Science and Technology, Wuhan, China, and written informed consent was obtained from the patient, in accordance with the Declaration of Helsinki. Peripheral blood mononuclear cells (PBMCs) were obtained from the patient (or her daughter) using Lymphocyte separation solution by the density gradient centrifugation at 700 g for 20 min. CD3+ T cells were isolated from the PBMCs by magnetic bead-conjugated anti-CD3 antibody (MiltenyiBiotec) and stimulated with anti-CD3/ anti-CD28 monoclonal antibody-coated magnetic beads（Thermo Fisher 11132D）in modified CTS™ OpTmizer™ T-Cell Expansion SFM (Thermo Fisher A10221-01) at 37 °C, 5% CO_2_ for 18-24 h. Cells were then separately transduced with different lentiviral vector encoding anti-CD19, or anti-CD20 or anti-CD22 single-chain variable fragment, linked with CD28 and 4-1BB costimulatory domains and CD3-ζ signaling domain (multiplicity of infection MOI=3), and cultured for 10-14 days in vitro. The medium was renewed every 2-3 days and cell concentration was adjusted to 1.5-2.0×10^6 /ml. The transduction efficiency and tumor cytotoxic effects of CAR T cells were monitored prior to infusion.

**Cell surface staining and flow cytometry**

For the analysis of CAR T cells, peripheral blood mononucleated cells were firstly stained with Alexa Fluor® 647 AffiniPure Goat Anti-Mouse IgG［F(ab')2 fragment specific］，then washed with PBS twice and stained with PERCP anti-human CD45 (BD Biosciences) and APC anti-human CD3 (BD Biosciences). The cells were then washed and resuspended in FACs buffer and analyzed by flow cytometry on the BD FACSCalibur system. The absolute count of CAR T cells in [peripheral blood](javascript:showjdsw('showjd_0','j_0')) =WBC * lymphocyte ratio * the proportion of CD3+ T cells in lymphocytes * the proportion of CAR T cells in CD3+ T cells.

**Copy number of CAR and cellular kinetics parameters**

CAR gene specific MGB probe was designed by Primer Express 3.0. Absolute quantification of CAR gene copy numbers was determined by droplet digital polymerase chain reaction (ddPCR) before and after infusion of CAR T cells. All the primers were synthesized by Invitrogen Company (Shanghai, China), and the sequences of the primers are listed in supplementary table 1. The cellular kinetic parameters (AUC0-28d, Cmax, Tmax, Tlast, T1/2) were assessed by noncompartmental methods using NonCompart package of R language.

Supplementary table 1. Sequences of primers used in ddPCR

| CD22 Forward | CTGCCGCGCCAGTCA |
| --- | --- |
| Reverse | CGGGCCGTTGCTGGTA |
| Probe | ACCATCTGGTCTTATCTG |
| CD19 Forward | CCGGCTGACCATCATCAAG |
| Reverse | GGTCTGCAGGCTGTTCATCTT |
| Probe | CAACAGCAAGAGCCAGG |
| CD20 Forward | CCAGGATCCTCCCCCAAA |
| Reverse | GGGACTCCAGAAGCCAGGTT |
| Probe | CCTGGATTTATGCCACATC |

**Detection of plasma cytokines**

Concentrations of plasma cytokines were analyzed on the FLEXMAP 3D system (Luminex xMAP Technology) using a Bio-Plex Pro Human Cytokine 48-Plex Screening Panel (Bio-Rad). Data were generated and analyzed by Bio-Plex Manager Software (Bio-Rad).

**Supplementary figure 1**

**A**  **B**

**
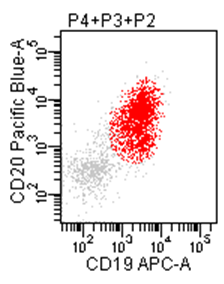

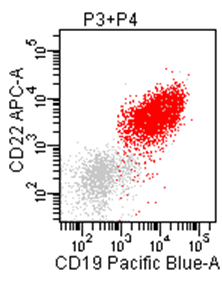
**

**C**  **D**


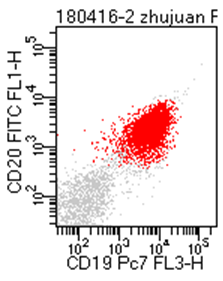

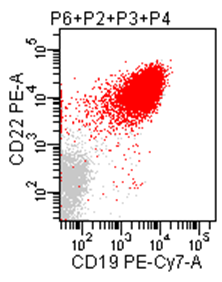


Supplementary Figure 1. The expression of CD19, CD20 and CD22 on lymphoma cells. A and B showed the expression of CD19, CD20 and CD22 in lymphoma cells before auto-CAR T cell therapy. C and D showed the expression of CD19, CD20 and CD22 in lymphoma cells before haplo-CAR T cell therapy.

| **Supplementary table 2. Timeline of treatment and efficacy of auto-CAR T** | | | | | | | | |
| --- | --- | --- | --- | --- | --- | --- | --- | --- |
| Time | Date | infusion | Therapy | WBC | IL-6 | ferritin | BM | PB |
| (day) |  | (×10^6/kg) |  | （×10^9/L） | (pg/ml) | (ug/L) | (%) | (%) |
| -27 | 3/12/2018 |  |  | 71.97 |  |  |  |  |
| -26 | 3/13/2018 |  |  |  |  |  | 68 |  |
| -25 | 3/14/2018 |  |  | 36.3 |  |  |  |  |
| -24 | 3/15/2018 |  |  | 33.34 |  |  |  |  |
| -23 | 3/16/2018 |  | VP-16 | 46.72 |  |  |  |  |
| -22 | 3/17/2018 |  | IFO | 44.34 |  |  |  |  |
| -21 | 3/18/2018 |  | VP-16 | 40.61 |  |  |  |  |
| -20 | 3/19/2018 |  | R^1^+IFO | 26.56 |  |  |  |  |
| -19 | 3/20/2018 |  | VP-16 | 20 |  |  |  |  |
| -18 | 3/21/2018 |  | I+IFO | 5.49 |  |  |  |  |
| -17 | 3/22/2018 |  | I+VP-16 | 5.92 |  |  |  |  |
| -16 | 3/23/2018 |  | I+IFO | 5.51 |  |  |  |  |
| -15 | 3/24/2018 |  | I+VP-16 | 4.26 |  |  |  |  |
| -14 | 3/25/2018 |  | I+IFO | 3.85 |  |  |  |  |
| -13 | 3/26/2018 |  | I | 3.6 |  |  |  |  |
| -12 | 3/27/2018 |  | R^1^+I | 3.35 |  |  |  |  |
| -11 | 3/28/2018 |  | I | 2.22 |  |  |  |  |
| -10 | 3/29/2018 |  | I | 2.8 |  |  |  |  |
| -9 | 3/30/2018 |  | I | 3.87 |  |  |  |  |
| -8 | 3/31/2018 |  | I |  |  |  |  |  |
| -7 | 4/1/2018 |  | I | 3.5 |  |  |  |  |
| -6 | 4/2/2018 |  | I |  |  |  |  |  |
| -5 | 4/3/2018 |  | F | 2.78 |  |  |  |  |
| -4 | 4/4/2018 |  | F | 2.94 |  |  |  |  |
| -3 | 4/5/2018 |  | F | 1.86 |  |  |  |  |
| -2 | 4/6/2018 |  |  | 1.45 |  |  |  |  |
| -1 | 4/7/2018 |  |  | 1.11 |  |  |  |  |
| 0 | 4/8/2018 | CART22 2 |  | 1.08 | 43.16 | 85 |  |  |
| 1 | 4/9/2018 | CART22 4 |  | 1.21 | 82.16 | 83.2 |  |  |
| 2 | 4/10/2018 | CART19 4 |  | 1.71 | 87.77 | 77 |  |  |
| 3 | 4/11/2018 | CART19 3 |  | 2.02 | 195.8 | 79.4 |  |  |
| 4 | 4/12/2018 |  |  | 2.37 | 217.5 | 92 |  |  |
| 5 | 4/13/2018 |  |  | 3.91 | 101.8 | 96.2 |  |  |
| 6 | 4/14/2018 |  |  |  | 92.43 | 123.9 |  |  |
| 7 | 4/15/2018 | CART22 4 |  | 6.88 | 52.72 | 130.2 |  | 51.4 |
| 8 | 4/16/2018 | CART22 2 |  | 11.41 | 57.03 | 139.3 |  |  |
| 9 | 4/17/2018 |  |  | 25.28 | 91.46 | 171.4 |  |  |
| 10 | 4/18/2018 |  | R^1^+I | 63.61 | 75.56 | 219.2 |  |  |
| VP-16: etoposide, 100 mg; IFO: ifosfamide, 1g; R: Rituximab, R^1^: 375mg/m^2^; | | | | | | | | |
| I: Ibrutinib 560 mg; F: Fludarabine 25mg/m^2^; WBC: white blood cell; BM:bone marrow; PB:peripheral blood. | | | | | | | | |

| **Supplementary table 3. Timeline of treatment and efficacy of haplo-CAR T cell therapy** | | | | | | | | |
| --- | --- | --- | --- | --- | --- | --- | --- | --- |
| Time | Date | infusion | Therapy | WBC | IL-6 | ferritin | BM | PB |
| (day) |  | (×10^6/kg) |  | （×10^9/L） | (pg/ml) | (ug/L) | (%) | (%) |
| -19 | 4/18/2018 |  | R^1^+I | 63.61 | 75.56 | 219.2 |  |  |
| -18 | 4/19/2018 |  | I | 44.53 |  |  |  |  |
| -17 | 4/20/2018 |  | I | 37.34 |  |  |  |  |
| -16 | 4/21/2018 |  | I | 44.25 |  |  |  |  |
| -15 | 4/22/2018 |  | I | 51.32 |  |  |  | 87.7 |
| -14 | 4/23/2018 |  | I | 62.78 |  |  |  |  |
| -13 | 4/24/2018 |  | R^2^+I | 75.55 |  |  |  |  |
| -12 | 4/25/2018 |  | I | 42.98 |  |  |  |  |
| -11 | 4/26/2018 |  | I+V | 5.91 |  |  |  |  |
| -10 | 4/27/2018 |  | I+V | 4.15 |  |  |  |  |
| -9 | 4/28/2018 |  | I+V | 4.95 |  |  |  |  |
| -8 | 4/29/2018 |  | I+V | 4.35 |  |  |  |  |
| -7 | 4/30/2018 |  | I+V | 6.21 |  |  |  |  |
| -6 | 5/1/2018 |  | R^2^+I+V | 7.75 |  |  |  |  |
| -5 | 5/2/2018 |  | I+V | 2.73 |  |  |  |  |
| -4 | 5/3/2018 |  | I+V+FC | 1.57 |  |  |  |  |
| -3 | 5/4/2018 |  | I+V+FC | 1.25 |  |  |  |  |
| -2 | 5/5/2018 |  | I+V+FC | 0.77 |  |  |  |  |
| -1 | 5/6/2018 |  | I+V | 0.47 |  |  |  |  |
| 0 | 5/7/2018 | CART22 2 |  | 0.7 | 27 | 489.5 |  |  |
| 1 | 5/8/2018 | CART22 4 |  | 0.38 | 7.26 | 451.5 |  |  |
| 2 | 5/9/2018 | CART22 2.5 |  | 0.58 | 7.66 | 469.6 |  |  |
|  | 5/9/2018 | CART20 2 |  |  |  |  |  |  |
| 3 | 5/10/2018 | CART20 4 |  | 0.55 | 10.61 | 434.2 |  |  |
| 4 | 5/11/2018 |  |  | 0.22 |  |  |  |  |
| 5 | 5/12/2018 |  |  | 0.04 | 92.91 | 415 |  |  |
| 6 | 5/13/2018 |  |  |  | 1314* | 575.6 |  |  |
| 7 | 5/14/2018 |  |  | 0.03 | 165.9* | 1666.6 |  | 0.1 |
| 8 | 5/15/2018 |  |  |  | 323 | 5360 |  |  |
| 9 | 5/16/2018 |  | I+V | 1.86 | 428.2 | 8186 |  |  |
| 10 | 5/17/2018 |  | I+V | 1.67 | 64.69 | 9474 |  |  |
| 11 | 5/18/2018 |  | I+V | 0.8 |  | 8078.7 |  |  |
| 12 | 5/19/2018 |  | I+V | 0.59 |  |  |  |  |
| 13 | 5/20/2018 |  | I+V | 0.6 |  | 3642 |  |  |
| 14 | 5/21/2018 |  | I+V | 0.66 |  |  | 0 | 0 |
| 15 | 5/22/2018 |  | I+V | 4.61 |  | 1930.5 |  |  |
| R: Rituximab, R^1^: 375mg/m^2^, R^2^: 500 mg/m^2^; I:Ibrutinib 560 mg; V:Venetoclax 400 mg; | | | | | | | | |
| F: Fludarabine 25 mg/m^2^; C: Cyclophosphamide 20 mg/kg; *: methylprednisolone 40 mg; WBC: white blood cell; BM:bone marrow; PB:peripheral blood. | | | | | | | | |
